# Supplementary figures and images for: Differential role of planar cell polarity gene Vangl2 in embryonic and adult mammalian kidneys
Source: PLoS One. 2020 Mar 23;15(3):e0230586. doi: 10.1371/journal.pone.0230586 (PMC7089571; doi:10.1371/journal.pone.0230586)

Derish et al, Sup Figure 1:  
Loss of Vangl2 does not disturb early nephrogenesis

A

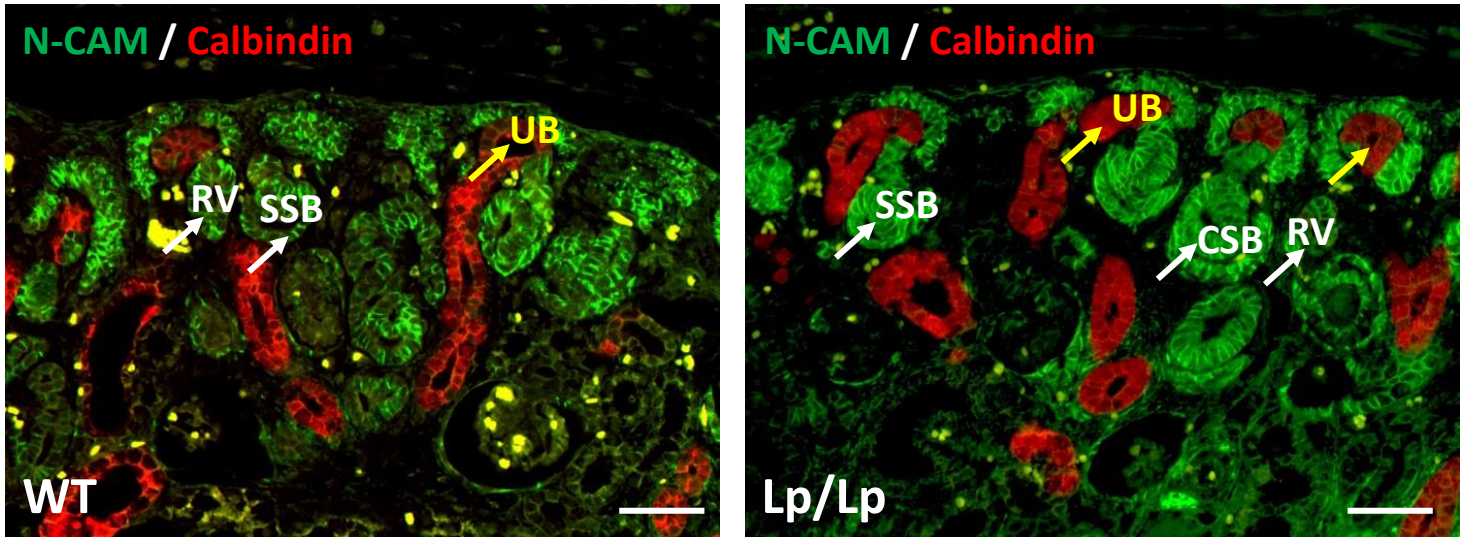

B

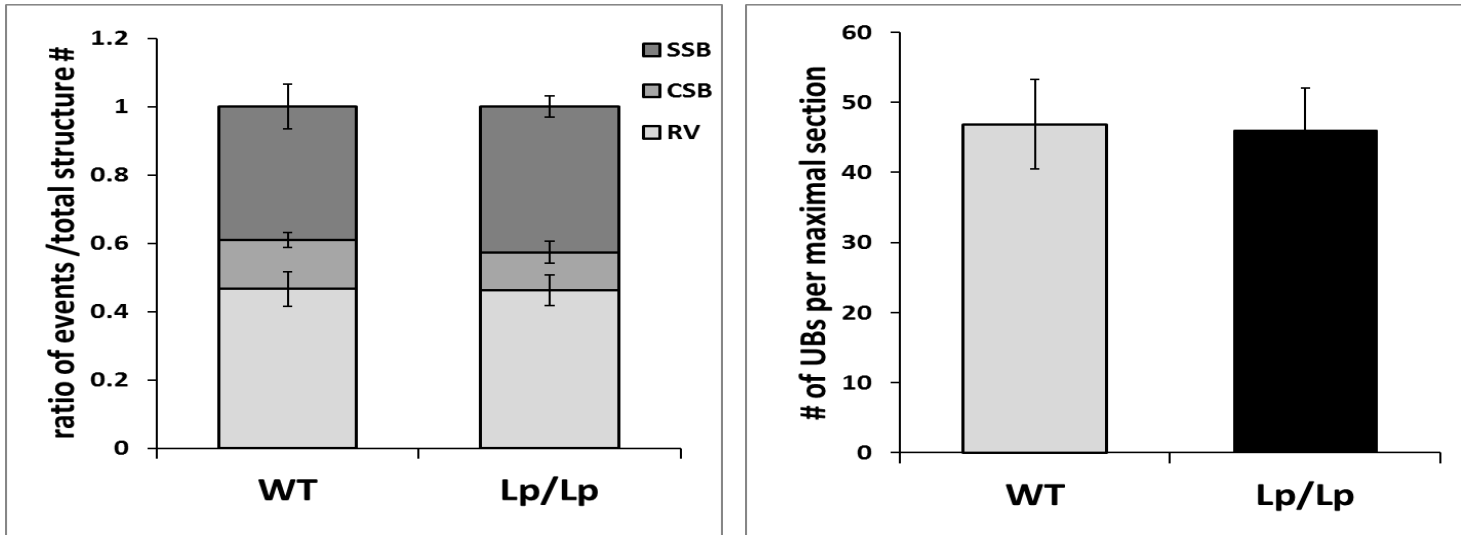

Supplement: S1 Fig — (A). Kidney development can be visualized using kidney sections. Examination of early nephrogenic structures such as renal vesicles (RVs), comma-shaped bodies (CSB) and S-shaped bodies (SSB) allows to define whether there are any defects in early nephrogenesis. These structures give rise to the glomerulus, proximal and distal tubules. RVs, CSBs and SSBs were detected with anti-NCAM antibody (green, white arrows). The collecting duct is derived from ureteric bud (UB) through repetitive ureteric bud branching. Each UB tip gives rise to a single nephron. The UB tips were visualized with anti-calbindin antibody (red, yellow arrows) in the E17.5 Vangl2Lp/Lp and wildtype E17.5 embryos. (B). The ratio of RV to SCB to SSB as well as the UB number were counted in the maximal kidney cross-sections; minimum 2 cross-sections per embryo per genotype were analyzed, 4 embryos per each genotype were assessed. ~ 300 nephrogenic structures and ~300 UB tips per genotype were analyzed. (PDF) [file pone.0230586.s001.pdf]
