# Supplementary material for: Differential role of planar cell polarity gene Vangl2 in embryonic and adult mammalian kidneys
Source: PLoS One. 2020 Mar 23;15(3):e0230586. doi: 10.1371/journal.pone.0230586 (PMC7089571; doi:10.1371/journal.pone.0230586)

Derish et al, Sup. Figure 2.
Morphological analysis of postnatal kidneys with conditional excision of Vangl2 compared to controls at postnatal days 1 and 7

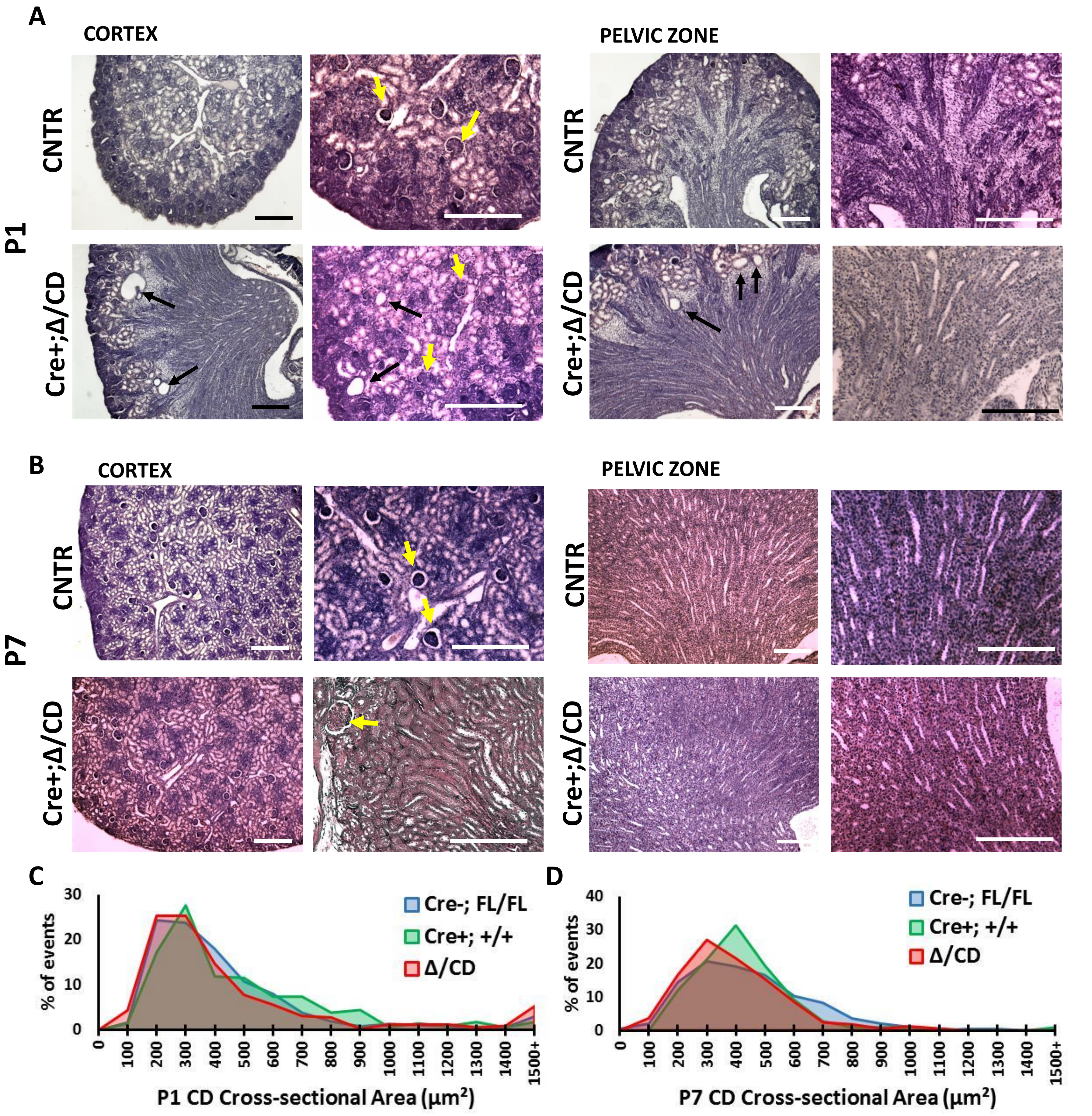

Supplement: S2 Fig — (A) P1 Vangl2Fl/Fl (control) and Cre(+);Δ/CD (Vangl2Δ/CD mutant) renal cortices and pelvic zones stained with hematoxylin and eosin (H&E); dilated tubules and cystic structures are indicated by black arrows, glomeruli are indicated by yellow arrows. Scale bars, 200 μm. (B) P7 Vangl2Fl/Fl and Cre(+);Δ/CD renal cortices and pelvic zones stained with H&E; glomeruli are indicated by yellow arrows. Scale bars, 200 μm. (C) Percentage of events within a given range of cross-sectional area in collecting duct tubules in P1 Vangl2Δ/CD (n = 362), Cre(-);Vangl2Fl/Fl (n = 324) and Cre(+);Vangl2+/+ (n = 337). (D) Percentage of events within a given range of cross-sectional area in collecting duct tubules in P7 Vangl2Δ/CD (n = 163), Cre(-);Vangl2Fl/Fl (n = 194) and Cre(+);Vangl2+/+ (n = 109). At least 3 animals per genotype were analyzed. (PDF) [file pone.0230586.s002.pdf]
