# Supplementary material for: Differential role of planar cell polarity gene Vangl2 in embryonic and adult mammalian kidneys
Source: PLoS One. 2020 Mar 23;15(3):e0230586. doi: 10.1371/journal.pone.0230586 (PMC7089571; doi:10.1371/journal.pone.0230586)

Derish et al, Sup. Figure 3:

Morphological analysis of postnatal kidneys with conditional excision of Vangl2 compared to controls at postnatal days 30 and 90

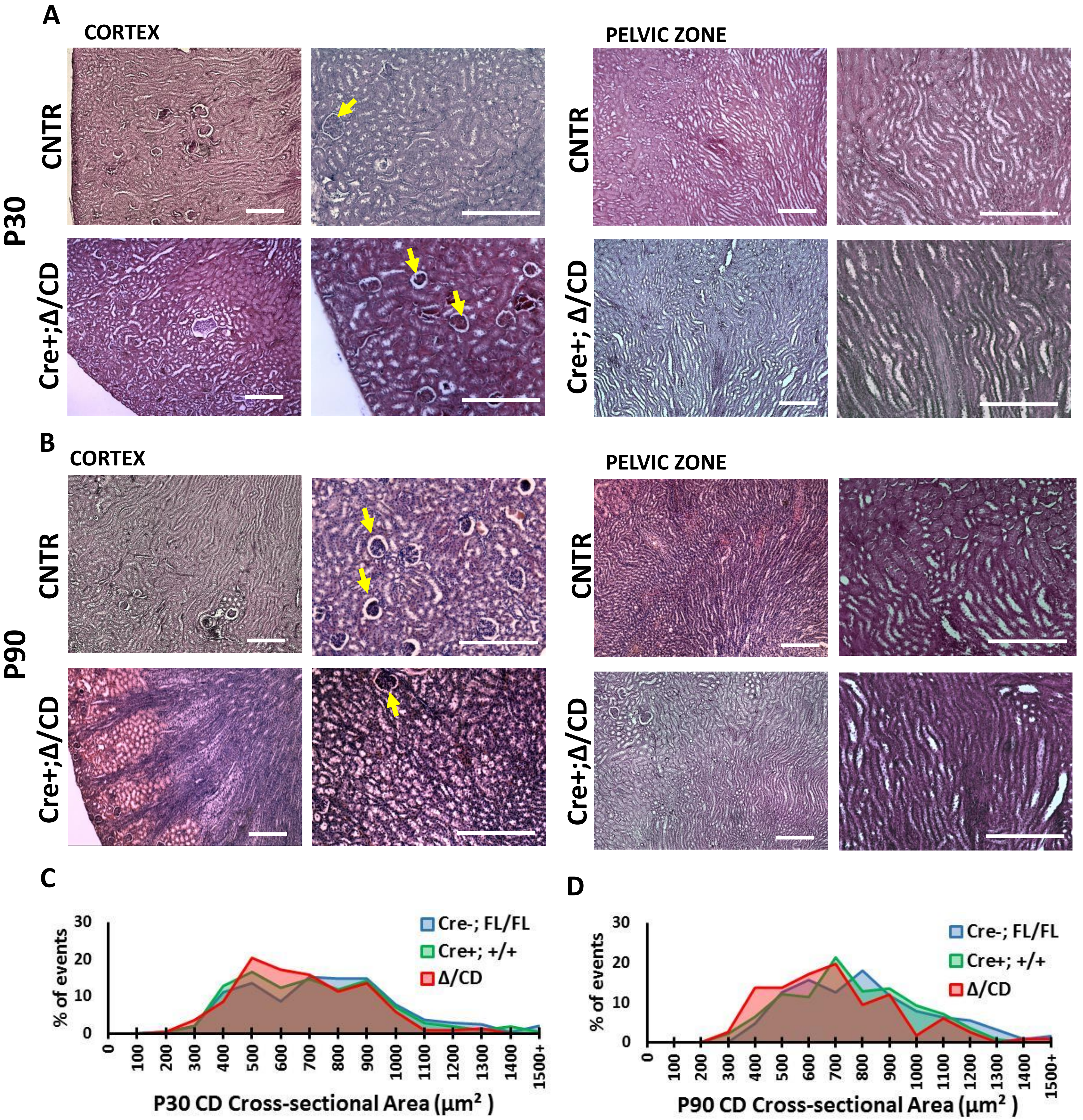

Supplement: S3 Fig — (A) P30 Vangl2Fl/Fl (control) and Cre(+);Δ/CD (Vangl2Δ/CD mutant) renal cortices and pelvic zones stained with H&E; glomeruli are indicated by yellow arrows. Scale bars, 200 μm. (B) P90 Vangl2Fl/Fl and Cre(+);Δ/CD renal cortices and pelvic zones stained with H&E; glomeruli are indicated by yellow arrows. Scale bars, 200 μm. (C) Percentage of events within a given range of cross-sectional area in collecting duct tubules in P30 Vangl2Δ/CD (n = 221), Cre(-);Vangl2Fl/Fl (n = 245) and Cre(+);Vangl2+/+ (n = 212). (D) Percentage of events within a given range of cross-sectional area in collecting duct tubules in P90 Vangl2Δ/CD (n = 117), Cre(-);Vangl2Fl/Fl (n = 128) and Cre(+);Vangl2+/+ (n = 141). At least 3 animals per genotype were analyzed. (PDF) [file pone.0230586.s003.pdf]
