# Supplementary material for: Differential role of planar cell polarity gene Vangl2 in embryonic and adult mammalian kidneys
Source: PLoS One. 2020 Mar 23;15(3):e0230586. doi: 10.1371/journal.pone.0230586 (PMC7089571; doi:10.1371/journal.pone.0230586)

Derish et al, Sup. Figure 4.

Morphological analysis of postnatal kidneys with conditional excision of Vangl2 compared to controls at 6- and 9 months.

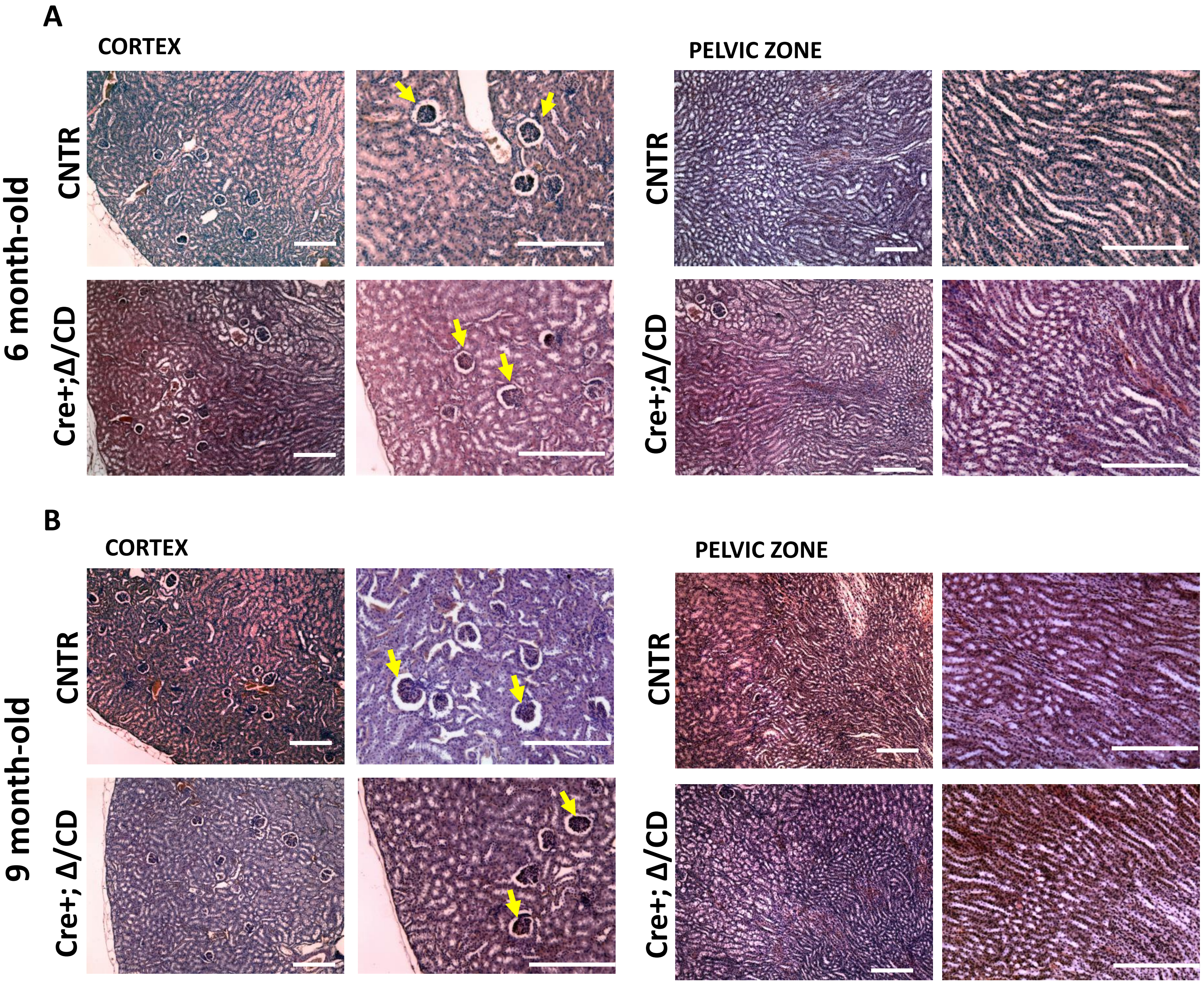

Supplement: S4 Fig — (A) 6 month-old Vangl2Fl/Fl (control) and Cre(+);Δ/CD (Vangl2Δ/CD mutant) renal cortices and pelvic zones stained with H&E; glomeruli indicated by yellow arrows. Scale bars, 200 μm. (B) P270 Vangl2Fl/Fl and Vangl2Δ/CD renal cortices and pelvic zones stained with H&E; glomeruli are indicated by yellow arrows. Scale bars, 200 μm. At least 3 animals per genotype were analyzed. (PDF) [file pone.0230586.s004.pdf]
