# Supplementary material for: Differential role of planar cell polarity gene Vangl2 in embryonic and adult mammalian kidneys
Source: PLoS One. 2020 Mar 23;15(3):e0230586. doi: 10.1371/journal.pone.0230586 (PMC7089571; doi:10.1371/journal.pone.0230586)

Derish et al, Sup Figure 5:
Morphological analysis of postnatal kidneys of conditional Pax2-Cre;Vangl2 mouse with Vangl2 excision in renal tubules.

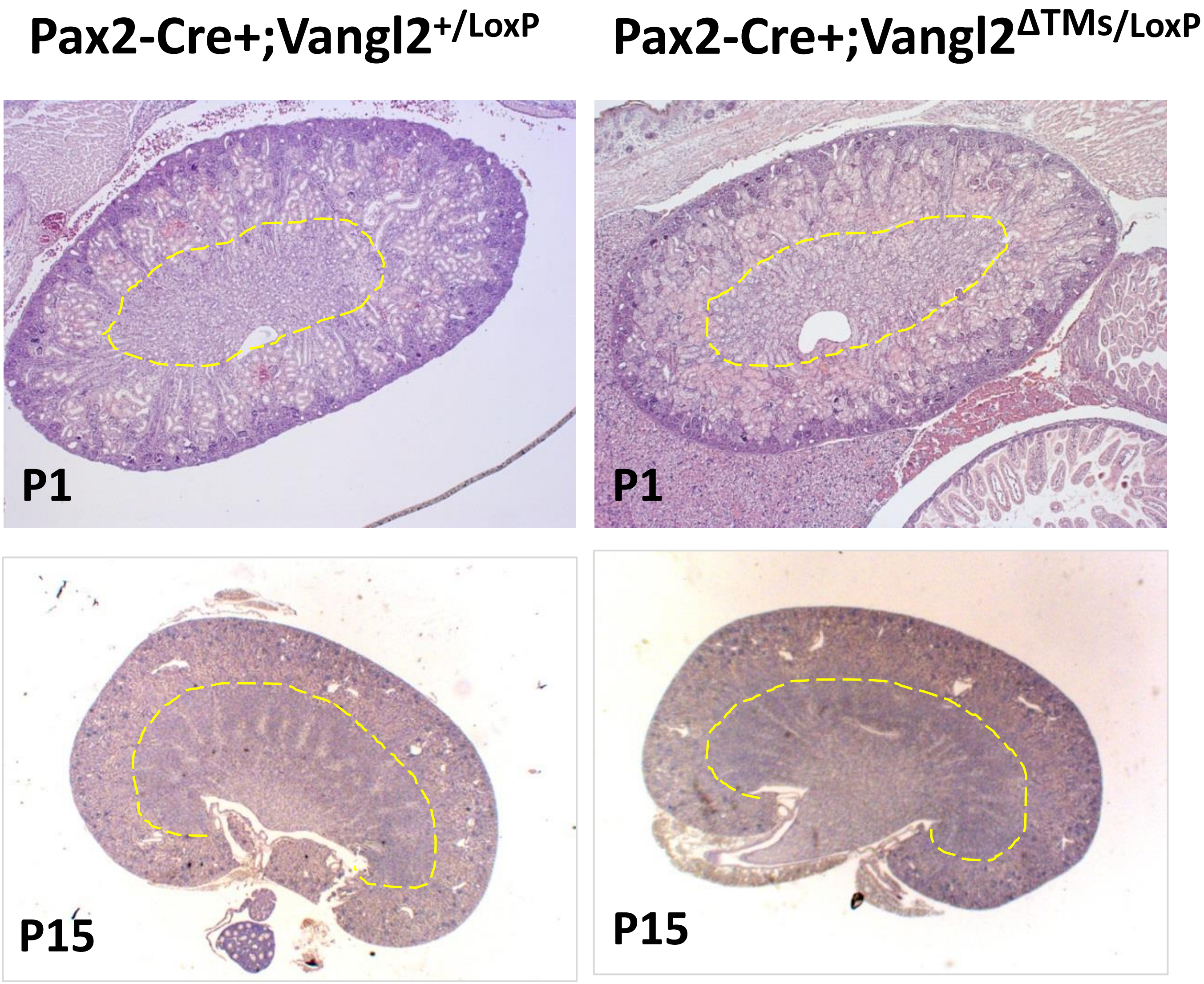

Supplement: S5 Fig — This mouse model was originally described in Copley et al, 2013. “ΔTMs” allele has a ubiquitous excision of Vangl2 exon 4 which encodes four transmembrane domains, TMs. The “LoxP” allele is the conditional excision of Vangl2 exons 2 and 3 encoding ATG translation initiation site. When bred to the Pax2-Cre deleter mouse, Cre-recombinase expressed under Pax2 promoter drives excision of Vangl2 exons 2 and 3 along the entire nephron. Medullary zone is demarcated by yellow line. The morphological analysis by H&E staining and light microscopy revealed no changes in the kidney architecture; no tubular dilatation or cysts were observed. 4 embryos per genotype were examined. (PDF) [file pone.0230586.s005.pdf]
