# Supplementary material for: Differential role of planar cell polarity gene Vangl2 in embryonic and adult mammalian kidneys
Source: PLoS One. 2020 Mar 23;15(3):e0230586. doi: 10.1371/journal.pone.0230586 (PMC7089571; doi:10.1371/journal.pone.0230586)

RAW IMAGES FOR FIG 2D

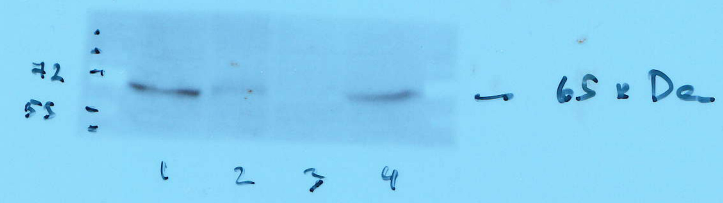

1- Primary CD cells Cre+; V2+/+
2- Primary CD cells Cre+; V2 Fl/D
3- E17.5 kidney Cre-; V2 D/D
4- E17.5 kidney Cre-; V2 Fl/Fl

gel 9.5%

Anti-Vangl2 (goat)
1: 1000

Donkey HRP-goat 1:10000
(in 5% milk buffer)

15 min (after suppl. wash).

P.S. Dilute HRP-goat in TBST)
1:10000

13/06/19

RAW IMAGES FOR FIG 2D

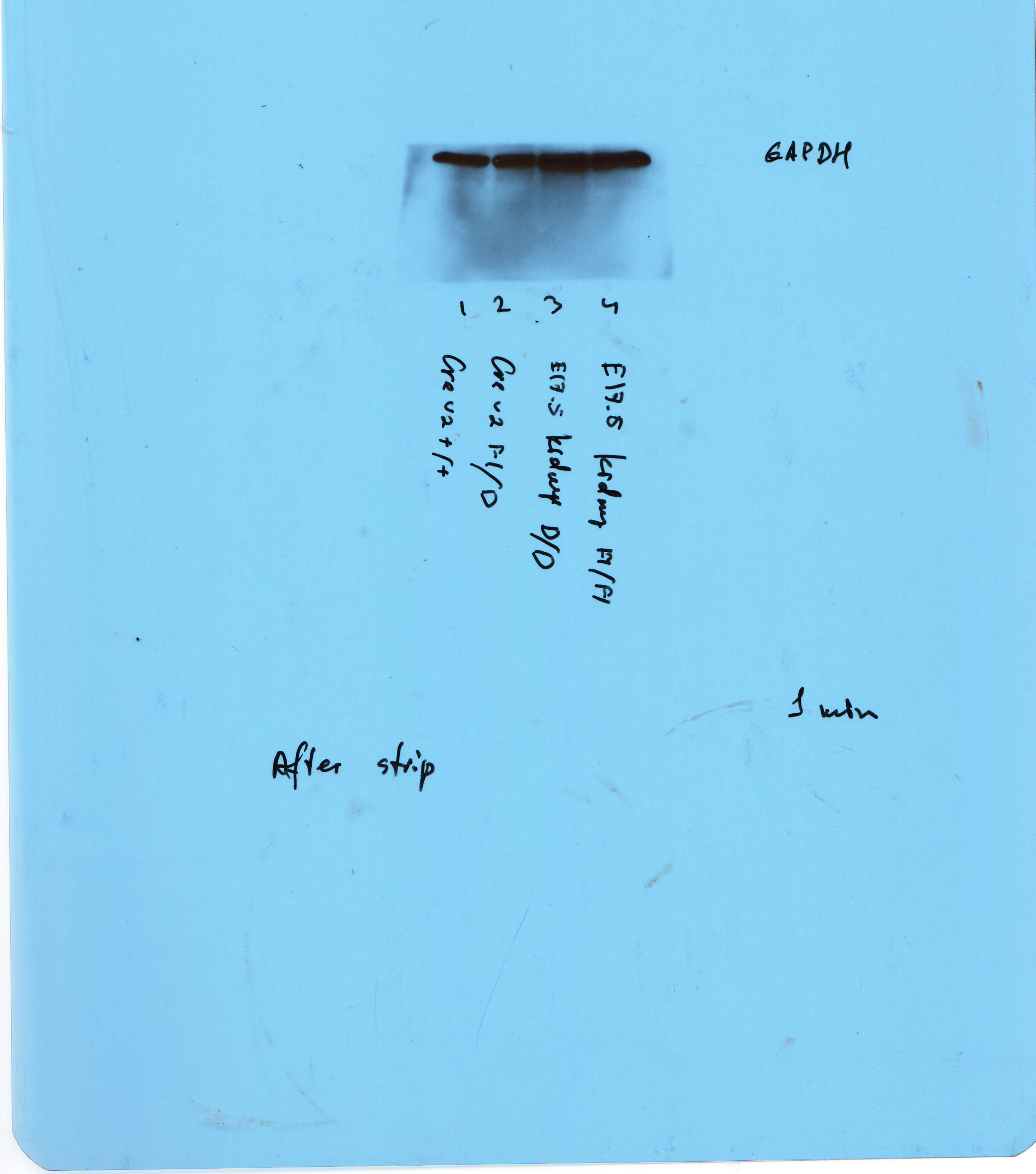

Supplement: S1 Raw images — (PDF) [file pone.0230586.s007.pdf]
