# Supplementary material for: Differential role of planar cell polarity gene Vangl2 in embryonic and adult mammalian kidneys
Source: PLoS One. 2020 Mar 23;15(3):e0230586. doi: 10.1371/journal.pone.0230586 (PMC7089571; doi:10.1371/journal.pone.0230586)
Supplement: Attachment — Submitted filename: Response to reviewers-Jan2020.pdf [file pone.0230586.s008.pdf]

Dear Dr. Shree Ram Singh

We would like to thank the reviewers for their thoughtful evaluation of our manuscript. We are pleased to see they concluded that our work was well-done and provided "in-depth" analysis of the issues in question. The reviewers made some valuable suggestions how to further improve the manuscript. Below, we provide point-by-point responses to the reviewers' suggestions. We trust that in its present form, our paper is suitable for publication in Plos One.

Reviewer 1:

1. *HoxB7-Cre mice used were on a mixed B16/CD-1 background. What is their cell/tissue expression pattern?*

The Hoxb7-Cre mouse was kindly provided by Dr. Carlton Bates (University of Pittsburgh, PA). This mouse expresses Hoxb7-Cre-EGFP transgene under regulation of the Hoxb7 promoter fragment extending from −1316 to 181bp of Hoxb7 gene. Dr. Bates extensively characterized expression of this transgene (Zhao et al, 2004, Developmental Biology, Ref # 29 in our revised manuscript): the transgene is expressed at highest levels in the nephric duct and branching ureteric bud throughout metanephric kidney development. Additionally, it was detected at high level in the midbrain-hindbrain junctions and a low level expression was seen in the dorsal root ganglia at E11.5. The same deleter mouse on the CD1 background was used by Dr. Goodyer's laboratory to excise the floxed EGFR gene (Zhang et al, JASN, 2010). We now state in the Materials and Methods "high efficiency of the Hoxb7-Cre allele on the CD1 was previously reported" and added this Reference to our manuscript – Reference #30).

2. *Related HoxB7-Cre mice seems to show low levels of expression in the dorsal root ganglia and the spinal cord? How did the authors address the issue?*

As mentioned by reviewer #1, Hoxb7 gene is expressed in DRG and spinal cord at low levels. High levels of Cre expression in the spinal cord of floxed Vangl2 mice could potentially have caused spinal cord defects. However, expression of the Bates'Hoxb7-Cre transgene in the spinal cord appears to be very low or undetectable (Zhao et al, 2004). Moreover, in our crosses, all Hoxb7-Cre;Vangl2$^{\Delta/CD}$ mice survived in predicted Mendelian proportions and had no signs of neural tube defects, indicating that the levels of Vangl2 expression in neural tissues were sufficient to allow normal development.

3. *Genotyping by visual inspection is not an ideal method due to variation in phenotypes. Proper PCR based Genotyping should be used.*

We agree with the reviewer #1 that, as a rule, genotyping by visual inspection is insufficient. In many instances (particularly at the start of the project), *Looptail* mice were genotyped by Sanger sequencing to confirm presence of the missense S464N mutation in Exon 8. The primers used to generate a PCR template and for nucleotide sequencing are now listed in the Materials and Methods. We used visual inspection since in our colony, both the homozygous

(craniorachischisis) and heterozygous (looped tail) phenotypes are 100% penetrant and concordant with the results of genotyping by sequencing.

4. *Immunofluorescence staining: Why was the thickness 30micrometer chosen for tile confocal? Does the author used 3D scan?*

We used confocal microscopy on 30 micron tissue sections to generate high resolution tile images of sagittal kidney sections. We did not generate 3D images. Instead, we used thick images to capture the long tubules which traverse large distances, particularly, in the kidneys from P30 and P90 animals. In order to develop this method, we experimented with various slice thickness and empirically found that the 30 micron tissue sections allow to visualize long tubular structures without a loss in the quality of antibody staining.

5. Statistical analysis: Does all the data pass Normality and Equal Variance test?

As suggested by reviewer # 1, we performed normality testing (Shapiro-Wilk test) and calculated the skewness for each dataset. These additional analyses revealed positive skew in all cases: the skew was larger in the embryonic and P1 samples comparing to the later postnatal stages, where the data distribution was more symmetric. The skew was presented graphically when relevant (Figures 1E,F; Figures 3F,G; Supplemental Figs 2,3). Our datasets were large (from ~ 80 to 300 individual data points for each time point and genotype); all compared samples were independent, had different spreads, the number of entry points and standard deviations. These features of datasets allowed us to perform the t-test assuming unequal variance, as opposed to equal variance (Student's t-test), which requires an equal number of entry points and symmetric data distribution. We modified the wording in the Materials and Method section accordingly.

6. Figure 5A: P30, & Figure 6C: E17.5, Statistical significance need to be analyzed again.

Prompted by the reviewer's comments, we re-analyzed and re-checked all datasets using unequal variance t-test. We made modifications in the graphs where appropriate:

1. Figure 1 D) *** changed to *.
2. Fig 5 A) In P1, * added between FLFL and D/CD.
3. Fig 5 A) In P7, * replaced by ** between FL/FL vs. d/CD.
4. Fig 5 A) In P90, * replaced by *** between FL/FL vs. d/CD.
5. Fig 5 B) In P1, *** replaced by ** between FL/FL vs d/CD.
6. Fig 5 C) In P1, ** replaced by *** between FL/FL vs. d/CD, and * put between ++ vs. d/CD

7. It's good to add some limitations for the study in the discussion so that the reader is able to see where the gaps are for this article and which the future work can focus on.

We now included some limitations in the Discussion (lines 473-486).

In response to the "minor" points, we formatted the revised manuscript to introduce line numbers, changed some wording and used abbreviations as recommended.

Reviewer #2.

We have made the necessary modifications according to suggestions of Reviewer #2: changed the words to depict black arrows in Supplemental Fig 2 and deciphered "UB" as "ureteric bud" at its first mention in the text.

We hope that with the revisions of our manuscript now meet journal standards and make it acceptable for publication in Plos One. We are looking forward to your decision.

Yours truly,

Elena Torban, PhD
